# Enzymatic and genetic characterization of lignin depolymerization by *Streptomyces* sp. S6 isolated from a tropical environment

**DOI:** 10.1038/s41598-020-64817-4

**Published:** 2020-05-08

**Authors:** Fatimah Azizah Riyadi, Analhuda Abdullah Tahir, Nurtasbiyah Yusof, Nurul Syazwani Ahmad Sabri, Megat Johari Megat Mohd Noor, Fazrena Nadia M. D. Akhir, Nor’azizi Othman, Zuriati Zakaria, Hirofumi Hara

**Affiliations:** 1 Department of Environmental Engineering and Green Technology, Malaysia-Japan International Institute of Technology, Universiti Teknologi Malaysia, Jalan Sultan Yahya Petra, 54100 Kuala Lumpur, Malaysia; 2 Department of Mechanical Precision Engineering, Malaysia-Japan International Institute of Technology, Universiti Teknologi Malaysia, Jalan Sultan Yahya Petra, 54100 Kuala Lumpur, Malaysia; 3 Department of Chemical Process Engineering, Malaysia-Japan International Institute of Technology, Universiti Teknologi Malaysia, Jalan Sultan Yahya Petra, 54100 Kuala Lumpur, Malaysia

**Keywords:** Environmental biotechnology, Applied microbiology

## Abstract

The conversion of lignocellulosic biomass into bioethanol or biochemical products requires a crucial pretreatment process to breakdown the recalcitrant lignin structure. This research focuses on the isolation and characterization of a lignin-degrading bacterial strain from a decaying oil palm empty fruit bunch (OPEFB). The isolated strain, identified as S*treptomyces* sp. S6, grew in a minimal medium with Kraft lignin (KL) as the sole carbon source. Several known ligninolytic enzyme assays were performed, and lignin peroxidase (LiP), laccase (Lac), dye-decolorizing peroxidase (DyP) and aryl-alcohol oxidase (AAO) activities were detected. A 55.3% reduction in the molecular weight (Mw) of KL was observed after 7 days of incubation with *Streptomyces* sp. S6 based on gel-permeation chromatography (GPC). Gas chromatography-mass spectrometry (GC-MS) also successfully highlighted the production of lignin-derived aromatic compounds, such as 3-methyl-butanoic acid, guaiacol derivatives, and 4,6-dimethyl-dodecane, after treatment of KL with strain S6. Finally, draft genome analysis of *Streptomyces* sp. S6 also revealed the presence of strong lignin degradation machinery and identified various candidate genes responsible for lignin depolymerization, as well as for the mineralization of the lower molecular weight compounds, confirming the lignin degradation capability of the bacterial strain.

## Introduction

Lignocellulosic biomass is the most abundant renewable organic carbon source on earth and can be effectively used as an alternative to biofuels, biochemicals, and biomolecules, with zero net carbon emission. However, the main challenge for its commercial application is the technology for converting lignocellulosic biomass into sugars for bioethanol/biochemicals. Lignocellulosic biomass, the main component of plant structure, mainly consists of two carbohydrate polymers, cellulose and hemicellulose, and an aromatic polymer, lignin, organized and interlinked together. Cellulose and hemicellulose are chains of polysaccharides that can be easily degraded by microbial enzymes or by chemical hydrolysis and are considered the main candidates for bioethanol and biochemical production. Meanwhile, lignin, the most complex among them, is a three-dimensional heterogeneous crosslinked macromolecule comprising numerous aromatic phenylpropanoid monomeric units identified as guaiacyl (G), p-hydroxyphenyl (H), and syringyl (S) units^1,2^. In the plant cell wall, lignin acts as a cellular glue between cellulose fibers and is covalently bonded with hemicellulose, thus enhancing its strength and rigidity. In addition, being aromatic in nature and relatively hydrophobic, lignin acts as an antimicrobial and waterproofing agent and provides carbohydrates protection from hydrolysis by microbial enzymes^3,4^.

There are several pretreatment approaches that use mechanical, chemical, physicochemical, and biological methods to depolymerize the recalcitrant lignin fraction of lignocellulosic biomass. Among them, biological pretreatment using microbial enzymes appears to be the most promising method, as it offers more environmentally friendly treatment with lower energy requirements and costs. In addition, biological pretreatment could offer advantages, as microbial degradation can be controlled to achieve desired, valuable aromatic and phenolic byproducts, such as vanillin, catechol, styrene, and polyphenols^2,5^. Ligninolytic microorganisms attack lignin by forming complex systems of oxidative enzymes^6^. Synergistic actions of groups of extracellular oxidative enzymes are required to initiate lignin depolymerization by generating highly reactive non-specific free radicals, which will simultaneously lead to cleavage of lignin inter-unit bonds (C-C and C-ether bonds) and further breakdown of the lignin structure^2,7^.

Some microorganisms, especially white-rot and brown-rot fungi, have been reported to depolymerize lignin during carbon limitation, secreting ligninolytic enzymes, such as lignin peroxidase (LiP), manganese peroxidase (MnP), versatile peroxidase (VP) and copper-containing laccase (Lac)^8–11^. However, most studies have not achieved commercial-scale lignin degradation since fungi have relatively complex genetic and protein expression characteristics^12^. Although less well identified and characterized than the ligninolytic enzymes from fungi, researchers have begun reporting some of the ligninolytic enzymes from bacteria^4,13–16^. However, actual lignin depolymerization and the relationship between the enzymology and molecular understanding of lignin degradation by bacteria are still poorly understood. Bacteria are predicted to possess other classes of ligninolytic enzymes that are not found in fungi. However, these potential ligninolytic bacteria are largely undiscovered, and many novel ligninolytic enzymes may still emerge. In addition, previous characterization studies on lignin-degrading bacteria mostly used lignin model dimers, such as β-aryl ether lignin^12^, veratrylglycerol-β-guaiacol ether^17^, guaiacylglycerol-β-guaiacyl ether^18^, and lignin-related aromatic acids^19^, to evaluate lignin degradation ability. However, depolymerization of polymeric lignin, which can closely resemble natural lignin, is limited and requires further evaluation.

As a tropical country, Malaysia provides a more stable growth temperature for microorganisms compared to temperate countries where the temperature fluctuates year-round. Microbes isolated from the local environment could improve biological treatment processes and further minimize the treatment cost for lignin degradation. In addition, the abundance of oil palm biomass in the country makes it a possible source of bacterial strains with lignin-degrading abilities. Therefore, in this study, biomass from a palm oil mill was used to isolate the lignin-degrading bacterium strain S6. Subsequently, the potential lignin-degrading ability of the strain was evaluated by performing enzymatic assays and gel-permeation chromatography (GPC) using Kraft lignin (KL) as the lignin model compound. The intermediate metabolites of the degraded lignin compounds were also identified using gas chromatography-mass spectrometry (GC-MS). Last, the draft genome sequence of the potential ligninolytic strain was obtained to confirm the degradation process and to reveal all the candidate genes responsible for lignin degradation.

## Results and Discussion

### Growth of *Streptomyces* sp. S6 on Kraft lignin

To identify the ability of the isolated bacterium to metabolize lignin, the growth of the bacterium on agar plates containing W-minimal media with 2.5 g/L KL was tested. In this study, we utilized KL as the lignin model. Strain S6 was successfully isolated from decaying oil palm empty fruit bunch (OPEFB) and grew well on the tested agar plate. The cells showed a morphology of clear white and leathery spores (Supplementary Fig. S1). To further confirm the ability of the strain to grow on lignin, the growth pattern was observed in liquid culture (in terms of optical density at 600 nm (OD_600_)) using 2.5 g/L KL as the sole carbon source (Supplementary Fig. S2). Although relatively slow, the growth of S6 in the presence of KL was clearly observed. The strain reached a maximum growth after 3 days of incubation, with an OD_600_ of approximately 0.2. This showed that despite being recalcitrant, this organism could adapt and metabolize KL as a carbon and energy source. In this study, KL was used as the sole carbon source without additional nutrients, to ensure that the bacterium can degrade lignin substrate. This could be the reason for the slow growth pattern of the bacterium. The organism was expected to depolymerize lignin and take up the lower molecular-weight KL-derived compounds and other organic compounds present in KL to grow.

Previous studies have reported that several lignin-degrading bacteria, including *Bacillus ligniniphilus* L1 and *Streptomyces* sp. F2621, require glucose, peptone, or yeast powder as additional carbon and nitrogen sources to stimulate bacterial growth on KL^20,21^. According to Zhu *et al*.^22^, an additional carbon source is necessary for microbial lignin degradation, even though the complete oxidation reaction of lignin is highly exothermic, i.e., generates energy, as the degradation of lignin is still too slow to function as the main source of metabolic energy for the microorganism. However, based on our results, strain S6 was able to grow even when KL was used as the sole carbon source, suggesting that this strain has the ability to degrade and use highly recalcitrant lignin as the carbon source. In addition, in a recent study, the growth of the lignin-degrading bacterium *R. opacus* was observed on plates after only two weeks of incubation with 2 g/L depolymerized softwood Kraft lignin (DL). *R. opacus* and *P. putida* EM42 growth was further evaluated in liquid cultures with 1 g/L DL, and maximum OD_600_ values of approximately 0.15 and 0.12, respectively, were observed after two weeks of cultivation, while the maximum OD_600_ value for non-depolymerized lignin was approximately one-third of that obtained with DL^23^.

### Analyses of extracellular ligninolytic enzyme activities

In most previous studies, lignin degradation by microorganisms were examined by evaluating the activities of ligninolytic enzymes secreted by the organisms. In addition to observing the growth pattern of strain S6, in this study, the activities of various reported ligninolytic enzymes, such as lignin peroxidase (LiP), manganese peroxidase (MnP), dye-decolorizing peroxidase (DyP), laccase (Lac) and aryl-alcohol oxidase (AAO), were also evaluated to confirm the lignin degradation ability of the strain. The cell-free supernatant was used as the crude enzyme to measure the activity of various extracellular ligninolytic enzymes. No MnP activity was detected in strain S6. On the other hand, considerable LiP (7.4 ± 0.032 U/L) and AAO activities (13.0 ± 0.170 U/L) were detected. However, in the case of Lac and DyP, S6 showed a low activity with 0.1 ± 0.095 U/L and 0.1 ± 0.036 U/L, respectively.

Previous enzymatic studies on numerous strong lignin degraders, such as *Pandoraea* sp. B-6, *Comamonas* sp. B-9, and *Novosphingobium* sp. B-7, reported that these strains only secrete MnP and Lac, and no obvious LiP activity was observed when strains were grown on KL^13,24,25^. In contrast, in this study, the highest enzyme activity was shown for LiP when grown in KL, and no MnP and slight Lac activities were detected. Previous observations for AAO activity were limited, but some fungi, such as *Aspergillus nidulans*^26^ and *Pleurotus ostreatus*^27^, and bacteria, such as *Sphingobacterium sp*. ATM^28^, were also reported to show AAO activity. According to Tamboli *et al*.^28^, AAO catalyzes aryl-α- and α-β-unsaturated γ-alcohol oxidation to the subsequent aldehydes with the simultaneous reduction of O_2_ to H_2_O_2_. The H_2_O_2_ generated is predicted to be used by ligninolytic peroxidases to regulate lignin degradation and to secrete enzyme activities.

### Evaluation of depolymerization activity by GPC analysis

Gel permeation chromatography (GPC) was performed to observe the degradation of the lignin polymer by comparing the changes in the molecular weight distribution of the polymer after being treated with the isolated bacterium. In this study, GPC was used to evaluate the molecular weight (Mw) of KL before and after 7 days of incubation with strain S6. Based on Table 1, the average Mw of KL on day 0 was approximately 3,401 Da. The Mw of KL decreased to 1,415 Da on day 3 and was further reduced to 1,286 Da after 7 days of being treated with strain S6, achieving 55.3% lignin degradation. In addition, the polydispersity (Mw/Mn) of lignin fragments decreased from 1.5 (control) to 1.1 (day 3 and day 7). A decrease in the molecular weight distribution of treated KL over time indicated that the KL polymer sample was successfully degraded after 7 days of treatment with S6. The bacterial strain showed fast depolymerization of KL after 3 days, as seen from the decrease in the molecular weight of KL and narrowing of the distribution ranges from the chromatograph (see Supplementary Figs. S3 and S4). This result is consistent with our growth curve result that demonstrates a higher growth curve during the first 3 days. Although fast depolymerization was observed after 3 days of treatment, we observed slight depolymerization with the continuation of 7 days of incubation, as seen from the shifted peak in the chromatograph (Supplementary Fig. S3). The presence of these shifted peaks indicates the production of smaller polymers of lignin as a result of bacterial growth. The GPC results suggest that this strain may be able to efficiently degrade polymeric lignin. We believed that strain S6 secretes ligninolytic enzymes that break the intermolecular C-C and C-O bonds of the lignin polymer into oligomers, trimers and dimers of lignin, indicating the feasibility of the biodegradation of the KL polymer. This result is parallel with previous reports on the bacterial utilization and depolymerization of lignin by GPC analysis^29–32^.

**Table 1:** Average molecular weights and polydispersity indices of Kraft lignin (KL) before (Control) and after treatment (Day 3 and Day 7) with strain S6.

Sample	Average Mw (Da)	Average Mn (Da)	Mw/Mn (polydispersity index)
Control	3,401.7 ± 59.2	2,285.7 ± 129.1	1.5 ± 0.1
Day 3	1,415.0 ± 35.9	1,331.0 ± 28.7	1.1 ± 0.0
Day 7	1,286.0 ± 17.2	1,222.3 ± 14.7	1.1 ± 0.0

### Identification of lignin monomers by GC-MS analysis

GC/MS has been proven to be a very suitable technique to analyze low molecular weight compounds (LMW) released from lignin, as reported by previous research^30,31,33,34^. The LMW compounds produced from KL were analyzed before and after 3 days and 7 days of treatment with strain S6, as summarized in Table 2 and Supplementary Fig. S5. Approximately eight aromatic compounds (1,4-dichlorobenzene, 2-methoxyphenol (guaiacol), vanillin, 1,3-dichlorobenzene, apocynin, 2,4-bis(1,1-dimethylethyl) phenol, bis(2-ethylhexyl) phthalate and triphenylphosphine oxide) and nine linear/branched oxygenated hydrocarbons (butyl acetate, 3-methylbutanoic acid, 2-methylbutanoic acid, 3-methyl 2 butanol, 1-4-dichloro benzene, 1-3-dichloro benzene, 2,6-dimethyldecane, 5-methyl-5-propylnonane, 4-methyltridecane, 4,6-dimethyldodecane, and bis(2-ethylhexyl) hexanedioate) were detected by GC-MS analysis. Several lignin monomeric compounds, such as 2,6-dimethyldecane, tridecane, 4-methyl, vanillin and apocynin, that were detected in the untreated KL samples were not detected in the KL samples treated with S6 on days 3 and 7. In addition, new monomer compounds, such as 3-methylbutanoic acid, 2-methylbutanoic acid, 1,4-dichlorobenzene, guaiacol, 5-methyl-5-propylnonane, and 4,6-dimethyldodecane, were detected on day 3 and/or day 7 after treatment with sample S6, suggesting that KL was further degraded by catalytic cleavage of C-C and C-O-C bonds. Since the first step of monomer production from lignin is depolymerization, it is important to break the bonds that hold the phenyl propane units. We believe that strain S6 produces several ligninolytic enzymes, such as peroxidases, that are responsible for depolymerizing lignin polymers and metabolizing low molecular weight aromatic compounds. In addition, the produced lignin fragments may be involved in different reactions, including hydrolysis of C4–ether bonds, dimethoxylation, aromatic ring opening, and C_α_–C_β_ breakdown^35^. Some linear alkanes, such as 2,6-dimethyldecane and nonane-5-methyl-5-propyl, were also detected, indicating that a ring-opening reaction occurred in strain S6. The phthalate derivative bis(2-ethylhexyl) phthalate has been detected and previously reported from fungal peroxidase degradation of lignosulfonate^36^ and from photodegradation of black liquor lignin^37^.

**Table 2:** GC-MS chromatographic peak identification of metabolites produced by the control (KL without strain S6) and strain S6 on day 0, day 3 and day 7 from KL degradation.

No.	RT	Identified compound	Strain S6		
			Control	D3	D7
1	4.223	butyl acetate	+	+	+
2	5.029	3-methyl-butanoic acid	−	+	+
3	5.350	2-methyl-butanoic acid	−	+	+
4	7.037	3-methyl-2-butanol	+	−	+
5	9.781	1,4-dichloro-benzene	−	−	+
6	9.781	1,3-dichloro-benzene	+	+	−
7	10.671	2,6-dimethyldecane	+	−	−
8	11.285	2-methoxyphenol (guaiacol)	−	−	+
9	14.596	5-methyl-5-propyl-nonane	−	−	+
10	14.597	4-methyl-tridecane,	+	−	−
11	14.598	4,6-dimethyl-dodecane,	−	+	−
12	16.461	vanillin	+	−	−
13	17.662	apocynin (acetovanillone)	+	−	−
14	17.936	2,4-bis(1,1-dimethylethyl) phenol	+	−	+
15	27.320	bis(2-ethylhexyl) hexanedioate	+	+	+
16	28.563	bis(2-ethylhexyl) phthalate	+	+	+
17	28.670	triphenylphosphine oxide	+	+	+

### Gene features of *Streptomyces* sp. S6

**Figure 1:**
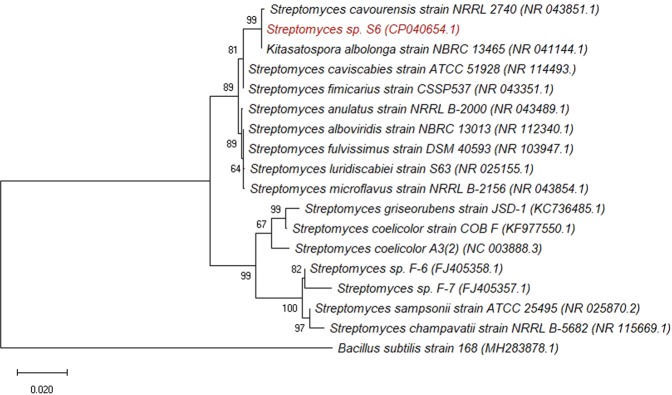
Phylogenetic analysis of *Streptomyces* strain S6 (highlighted) with the related species strains based on 16S rRNA gene homology. This tree was generated by the maximum-likelihood algorithm using Jukes-Cantor distance correction and the bootstrap resampling method after 500 replications, which was conducted with MEGA 7 software. GenBank accession numbers of related species are shown in parentheses. *Bacillus subtilis* strain 168 (MH283878.1) was used as an outgroup to root the tree.

The draft genome of strain S6 was 6,420,514 bp in size, with a GC content of 71.23%, 9,405 coding sequences (CDS), 6,064 proteins with predicted functions, and 3,341 hypothetical proteins (Table 3). Strain S6 was classified as a gram-positive bacterium, *Streptomyces sp*., according to its morphological characteristics, 16S rRNA region, and complete genome sequences. Based on the 16S rRNA sequence phylogenetic tree (Fig. 1), strain S6 is closely related to other *Streptomyces* groups, showing the highest similarity to *Streptomyces cavourensis* strain NRRL 2740 (NR_043851.1) and *Kitasatospora albolonga* strain NBRC 13465 (NR_041144.1), a homotypic synonym of *Streptomyces albolongus*. From the phylogenetic tree, *Streptomyces coelicolor* A3(2) (NC_003888.3)^38^, *Streptomyces* sp. F-6 (FJ405358.1) and *Streptomyces* sp. F-7 (FJ405357.1)^16^, as well as *Streptomyces griseorubens* strain JSD-1 (KC736485.1)^39^ were also previously reported to demonstrate lignin-degrading ability. A gram-positive *Bacillus subtilis* strain 168 (MH283878.1), was used as the outgroup strain to root the phylogenetic tree. The protein-coding genes from the draft genome sequences of strain S6 were annotated and classified into 23 and 27 functional classes/subsystems according to COG and RAST, respectively. Based on the COG functional categories (Supplementary Table S1), general processes and metabolic pathways related to amino acids, carbohydrates, fatty acids, and lipids were dominant. In addition, the moderate quantity of categories, such as secondary metabolite biosynthesis, transport, and catabolism in the subsystem features suggests that strain S6 is capable of surviving and metabolizing lignin or aromatic compounds.

**Table 3:** Genome features of *Streptomyces* sp. strain S6.

Attribute	Value
Genome size (bp)	6,420,514
GC content	71.23
Contigs	3,896
Contig L50	904
Contig N50	2,057
RNAs	41
Number of coding sequences	9,405
Proteins with predicted functions	6,064
Hypothetical proteins	3,341

### Identification of ligninolytic enzyme genes through the draft genome sequence of strain S6

Lignin degradation that occurs in nature is mainly a result of two processes: first is the depolymerization of native polymeric lignin to produce low molecular weight aromatic compounds, followed by the mineralization of the resulting aromatics. Depolymerization of native lignin is driven by extracellular oxidative enzymes, such as Lip, MnP, and Lac, which have been highly reported in fungi. Bacteria are thought to have a lesser amount of these powerful ligninolytic enzymes and are generally predicted to play a key role in the second stage of lignin degradation: the mineralization of lignin-derived aromatic compounds. Although studies on the enzymology of lignin-degrading bacteria are still limited, bacteria are also expected to use extracellular peroxidases to initiate lignin depolymerization, and these enzymes are still used as indicators of bacterial lignin degradation. The draft genome of strain S6 was used to verify the presence of genes that encode known ligninolytic enzymes. Since no MnP activity was detected in this study, it was not surprising that searching for the MnP gene in strain S6 also showed the absence of the gene. However, when comparing strain S6 with the reference genome that encodes LiP and Lac, only open reading frames (ORFs) with very low amino acid identities were found: ~32% against LPOA of *Phanerochaete chrysosporium* (UniProt Entry: P06181) and ~34% against lccA of *Haloferax volcanii* strain ATCC 29605 (UniProt Entry: D4GPK6).

The absence or low enzymatic activities detected for some of the enzymes could be due to the inadequacy of the reaction conditions and substrate concentration used in the reported enzymatic assays^40–43^; therefore, further optimization is required in the next studies. However, in a previous study, Shi *et al*.^44^ also reported that although Lac and MnP activities were detected for *Cupriavidus basilensis* B-8, no MnP or Lac genes were found in the strain. According to Davis *et al*.^45^, homologs of the most common ligninolytic peroxidases, such as LiP and MnP, have not been fully acquired in biochemical studies on lignin-degrading bacteria, and analyzing the gene sequences or proteomes of ligninolytic bacteria revealed no homologs. In addition, previous studies on lignin degradation were mostly conducted on fungi. Thus, the well-known ligninolytic peroxidases, the Class II plant peroxidase superfamily, are only limited to fungi, but bacteria are expected to possess unique lignin-degrading mechanisms and other types of peroxidases^46^. Surprisingly, our analysis showed that some genes in S6 demonstrated high homology and similarity with genes encoding DyP in the reference genome, with 59% similarity to the DyP of *Rhodococcus jostii* RHA1 (UniProt Entry: Q0SE24) and 44% similarity to the Tfu_3078 gene of *Thermobifida fusca* strain YX (UniProt Entry: Q47KB1). These results are consistent with the DyP activity detected, although at low quantity. Variations in the genes and activities of enzymes secreted by S6 in comparison to previous reports could indicate that strain S6 possesses a diverse regulation mechanism for novel ligninolytic enzyme-encoding genes.

### Putative genes responsible for lignin degradation and central intermediate metabolic pathways based on draft genome sequences

The complex structure of lignin requires the synergistic action of various lignin-degrading enzymes. Exploring the draft genome of strain S6 revealed the presence of putative genes that could be responsible for the degradation of lignin and lignin-derived aromatic compounds (Supplementary Table S2). Peroxidases are reported to be the key enzymes in lignin degradation and could be involved in the initial depolymerization stage^47,48^. The draft genome of strain S6 showed the presence of bacterial peroxidases, such as catalase, catalase-peroxidase, peroxiredoxins, glutathione peroxidase, and DyP-type/ deferrochelatase peroxidase, which may be responsible for the enzymatic activities observed during the peroxidase assay. In a previous study on bacterial lignin degradation, DyP appeared to have wide substrate specificity and was shown to degrade not only high redox anthraquinone dyes and aromatic sulfides^49^ but also veratryl alcohol, phenolic/nonphenolic lignin compound units^9,17^, and manganese^17^. In addition, DyP was also reported to degrade the aryl ether bonds in lignin model compounds^12,50^. Catalase peroxidase was reported to degrade a phenolic lignin model compound^46^. Peroxiredoxin is a cysteine-dependent peroxidase that reacts with hydrogen peroxide, organic hydroperoxides, or peroxynitrite. Peroxiredoxin is critical in bacterial cells and acts as an antioxidant defense system to protect cellular components from oxidative damage while regulating various signaling processes, such as cell proliferation, reactive oxygen species scavenging, and cell death^51^.

Several oxidoreductases, ferroxidases (including multicopper oxidases), oxidases, reductases, and dehydrogenases were also present in the genome of strain S6. These enzymes might also be responsible for the AAO activity of strain S6. The oxidases function as auxiliary enzymes by generating hydrogen peroxides utilized by peroxidases for the degradation of lignin and aromatics^52^. The oxidoreductases degrade lignin and aromatic compounds by generating nonspecific free radicals and reactive intermediates. Thus, for cell survival, these intermediates need to be removed or transformed into more stable and less toxic compounds^53^. In addition, multicopper oxidase, which was reported as a bacterial laccase, was also detected. Thus, the laccase activity observed in this study could be due to the presence of this enzyme. Multicopper oxidase functions by reducing dioxygen to water while oxidizing phenolic and nonphenolic compounds to their respective radical species, which can undergo further hydration, oxidation, and polymerization/depolymerization reactions^54,55^. Dehydrogenase works in the lignin mineralization stage by cleaving ether linkages and targeting toxic aldehydes, converting them into stable intermediates intracellularly^56^. According to Sato *et al*.^57^, alcohol dehydrogenase, when combined with short-chain dehydrogenases/reductases and glutathione S-transferase, can synergistically degrade ether linkages of the lignin model compound.

The draft genome analysis of strain S6 also revealed several proteins related to the oxidative stress response for protection from reactive species and detoxification mechanisms during aromatic metabolism, including catalase, superoxide dismutase, glutathione, glutaredoxins, peroxiredoxins, and thioredoxin. Cytochrome P450-related genes, a superfamily of heme-thiolate proteins, were also detected in the draft genome of S6. Cytochrome P450 detected in S6 was also previously reported to support lignin degradation by acting as an oxidase enzyme and was also found to catalyze several enzymatic reactions for the conversion of aromatic/xenobiotic chemicals into more polar and/or less toxic derivatives^58,59^. In addition, lignin degradation by fungi requires the support of the quinone oxidoreductase system to utilize Fenton chemistry for degradation^60^. Thus, the presence of quinone oxidoreductase genes in strain S6 could also indicate its lignin-degrading ability. In previous reports, NADPH:quinone oxidoreductase was expressed by the bacterium *Pandoraea* ISTKB and fungus *Trametes versicolor*, and the gene was reported to degrade lignin by the Fenton reaction^61,62^. Some transferase and hydratase enzymes, such as acetyl-CoA acetyltransferase and enoyl-CoA hydratase, were also reported to have roles in the degradation of the aromatic compound benzoate^63^.

Many studies have reported that bacteria take part in the mineralization of lignin-derived low-molecular-weight aromatic compounds. These aromatic compounds generally have restricted chemical reactivity and are commonly attacked with the help of oxygen by oxygenase enzymes^64,65^. Such limited reactivities also lead to the production of some common central intermediates, such as catechol, protocatechuate, gentisate or homogentisate, that will further undergo central-ring cleavage catalyzed by ring-cleaving dioxygenase. Detection of these metabolic pathways in strain S6 confirmed the potential of this strain for lignin degradation. Metabolic pathway analysis with the RAST subsystem and KEGG revealed the genes involved in the metabolism of central aromatic intermediates (Supplementary Table S3). The draft genome of S6 revealed genes responsible for the catechol branch of the beta-ketoadipate and homogentisate pathways as well as salicylate and gentisate catabolism, including catechol 2,3-dioxygenase, homogentisate 1,2-dioxygenase, 4-hydroxyphenylpyruvate dioxygenase, and fumarylacetoacetase, which are involved in the degradation of central intermediate pathways.

## Materials and Methods

### Bacterial growth on Kraft lignin

Sample preparation and lignin-degrading bacterial isolation were performed according to Tahir *et al*.^66^. Isolated bacteria were grown in 100 mL of Luria Bertani (LB) broth for cell enrichment and incubated at 30 °C with shaking at 160 rpm until OD_600_ ~ 1.0 was reached. After that, a 50 mL aliquot of each bacterial strain was centrifuged at 10,000 rpm for 10 min to pellet the cells. The bacterial cells were washed twice with W-minimal media to remove LB broth completely and resuspended in 50 mL of W-minimal media to be used as a seed culture for KL degradation. The washed cells were inoculated into W-minimal media with KL at the initial OD_600_ ∼ 0.1 in a 150 mL flask and incubated at 30 °C and 160 rpm for 10 days. KL was used as the sole carbon source. The medium without seed culture was used as a control. One milliliter of the sample was taken daily to evaluate bacterial growth. Lignin is oxidized during the growth conditions, and the color of KL is dark, which could considerably interfere with the absorbance measurements. Thus, the culture sample was centrifuged, and the obtained cells were diluted with W-minimal media, vortexed and used to measure the OD_600_.

### Ligninolytic enzyme activity

Collected samples were centrifuged at 10,000 rpm for 5 min to separate the supernatant from the cell pellets. The cell-free supernatant was collected and used as a crude enzyme to measure ligninolytic enzyme activity. LiP enzyme activity was measured following veratryl (3,4-dimethoxybenzyl) alcohol oxidation to veratryl aldehyde^40^. The enzyme reaction consisted of 500 µL of 100 mM sodium tartrate buffer (pH 3.8), 500 µL of 4 mM veratryl alcohol, and 0.1 mL of crude enzyme. To start the reaction, 0.1 mL of H_2_O_2_ (2 mM) was added to the mixture, and incubated at 30 °C for 5 min. A change in absorbance at 310 nm (ɛ = 9,300 M/cm) was observed. MnP activity was determined based on phenol red assay^41^ by mixing 0.0025% phenol red, 50 mM sodium tartrate buffer (pH 4.5), 0.2 mM MnSO_4_, 0.1 mM H_2_O_2_, and crude enzyme in a total volume of 1 mL. The enzyme mixture was incubated at 30 °C for 5 min and monitored at 431 nm (ɛ = 22,000 M/cm). Lac enzyme activity was assayed through guaiacol oxidation^42^. The enzymatic mixture consisted of 2 mM guaiacol, 10 mM acetate buffer (pH 5.0), and crude enzyme to a total volume of 1 mL. Guaiacol oxidation was determined by incubating the mixture at 25 °C for 2 hours, and the increase in the absorbance at 450 nm (ɛ = 12,100 M/cm) was observed. DyP activity was determined by monitoring the anthraquinone dye decolorization^43^ Reactive Black 5 (RB5) at 597 nm (ɛ = 37,000 M/cm). The enzymatic reaction consisted of 25 mM citrate buffer (pH 3) and crude enzyme at a pH of 3.2, followed by the addition of 119 µM RB5. The reaction was started by adding 0.3 mM H_2_O_2_ at 30 °C. The lignin-degrading auxiliary enzyme activity of AAO was also determined by observing veratryl (3,4-dimethoxybenzyl) alcohol oxidation to veratryl aldehyde at 310 nm (ɛ = 9,300 M^−1^ cm^−1^)^67^. The enzymatic reaction consisted of 5 mM veratryl alcohol, 0.1 M sodium phosphate buffer (pH 6.0), and crude enzyme. All enzymatic assays were conducted in triplicate.

### Characterization of lignin-degrading activity by GPC and GC-MS analysis

The KL molecular weight (Mw) distribution before and after pretreatment with strain S6 was determined using aqueous GPC (LC-20AD GPC Shimadzu, Japan), and the procedure was performed using the acetobromination method^68^ and following the method by Tahir *et al*.^66^. Briefly, tetrahydrofuran (THF) (HPLC grade, without stabilizer) was used as the mobile phase, and Styragel HR-5E, as well as Styragel HR-1 (Waters, Milford, MA, USA), were used as the separation columns. System calibration was performed using several polystyrene standards (Supplementary Fig. S4) and detected by UV at a wavelength of 280 nm. Each analysis was performed in triplicate. The degradation products were detected using GC-MS analysis according to Tahir *et al*.^66^. Briefly, once the culture in LB medium reached OD_600_ ~ 1.0, the cells were harvested by centrifugation at 10,000 × *g* for 10 min and resuspended twice in 100 mL of W-minimal media, prior to washing. Then, 10 mL of W-minimal media and 2.5 g/L KL were added to the cells for seven days, and the cells were incubated at 30 °C with shaking at 160 rpm. Aliquots (1 mL) of each culture were collected every 24 hours and used as the sample. The identification of intermediate metabolites of lignin-derived compounds from the isolate was performed by comparing the mass spectra to the National Institute of Standards and Technology 11 (NIST 11) library provided with the instrument.

### Draft genome sequencing and functional annotation

The genomic DNA of S6 was extracted prior to draft genome sequencing using the QIAamp DNA Mini Kit (Qiagen). The draft genomic libraries were sequenced on the Ion X5 XL sequencer system (Thermo Fisher Scientific). Reads from the Ion X5 XL sequencer were quality trimmed using CLC Genomics Workbench software (version 11.0.1; CLC bio, Aarhus, Denmark). High-quality reads were *de novo* assembled using the same software. The sequences and homologous sequences obtained from GenBank were aligned with the Clustal W 2.0 algorithm. Phylogenetic analysis was performed with the maximum-likelihood algorithm using Jukes-Cantor distance correction and the bootstrap resampling method from the software package MEGA 7^69^. The 16S rRNA gene sequence of *Bacillus subtilis* strain 168 (MH283878.1), taken from GenBank, was used as an outgroup to root the tree. The genome sequences of bacterial strains were functionally annotated using the Rapid Annotation using Subsystem Technology (RAST) database. Protein-coding genes were also annotated and grouped based on the functional classes using the Cluster of Orthologous Groups (COG) and PATRIC databases. Genes related to lignin degradation in strain S6 were identified using the KEGG database and manually annotated by performing a BLASTP search against the ‘nr’ database. Genes involved in the metabolism of central aromatic intermediates were identified using the RAST subsystem features.

## Conclusion

This study demonstrated that a *Streptomyces* sp. isolated from a tropical environment could be a useful choice for lignin degradation. The strain was successfully proven to possess KL-degrading ability, while producing various low molecular weight lignin-derived compounds, and significant enzymatic activities (LIP, LAC and AAO). Comprehensive draft genomic analysis of *Streptomyces* sp. S6 revealed various candidate genes related to lignin degradation and potential degradation pathways. To fully understand the metabolic characteristics of lignin and to identify the actual enzymes that are responsible for lignin degradation by strain S6, more experiments and investigations, such as gene expression and proteomics, are needed in the future. Since the bacterial lignin depolymerization mechanism has not been fully understood, a study that combines both characterization and genomic assays using KL as the lignin model is highly important and can have an impact on the conversion of lignin into renewable chemicals.

## Supplementary information

Supplementary Information.

## Data Availability

All data generated or analyzed during this study are included in this published article (and the Supplementary Information files). The Whole Genome Shotgun (WGS) project used in this paper has been superseded by the complete genome in (non-WGS) GenBank record CP040654 (https://www.ncbi.nlm.nih.gov/nuccore/CP040654). The version described in this paper is version SDIJ01000000, which consists of sequences SDIJ01000001-SDIJ01003896.
