# Supplementary material for: Enzymatic and genetic characterization of lignin depolymerization by Streptomyces sp. S6 isolated from a tropical environment
Source: Sci Rep. 2020 May 8;10:7813. doi: 10.1038/s41598-020-64817-4 (PMC7210275; doi:10.1038/s41598-020-64817-4)
Supplement: Supplementary file 1 — Supplementary Information. [file 41598_2020_64817_MOESM1_ESM.pdf]

Supplementary Information:

Enzymatic and genetic characterization of lignin depolymerization by *Streptomyces* sp. S6 isolated from a tropical environment

Fatimah Azizah Riyadi §, Analhuda Abdullah Tahir §, Nurtasbiyah Yusof, Nurul Syazwani Ahmad Sabri, Megat Johari Megat Mohd Noor, Fazrena Nadia MD Akhir, Nor'azizi Othman, Zuriati Zakaria, and Hirofumi Hara*

Supplementary information content:

Table S1. Classification of predicted proteins in subsystem features and their distribution in various functional groups shown in strain S6

Table S2. Annotated genes encoding ligninolytic degrading enzymes in strain S6

Table S3. Putative genes involved in metabolism of central aromatic intermediates

Fig. S1. Growth of isolated strain S6 in solid media containing alkali lignin as sole carbon source

Fig. S2. Growth curve of strain S6 in liquid medium containing alkali lignin as sole carbon source. A non-inoculated control is shown with a square marker line.

Fig. S3. GPC chromatogram data of the isolated strain S6 at (i) Control (untreated KL without S6), (ii) day 3 and (iii) day 7.

Fig. S4: GPC chromatogram illustrating the lignin standard markers of the (a) calibration line and (b) molecular weight (1): 193 000 Da, (2): 16 200 Da, (3): 2 620 Da, (4): 953 Da, (5): 533 Da

Fig. S5. A: TIC of metabolites produced by control (untreated samples) B: TIC of metabolites produced by strain S6 from KL degradation (a) day 0, (b) day 3, and (c) day 7

Table S1. Classification of predicted proteins in subsystem features and their distribution in various functional groups shown in strain S6

Class	Value	Percentage	Description
J	130	1.38	Translation, ribosomal structure and biogenesis
A	1	0.01	RNA processing and modification
K	91	0.97	Transcription
L	91	0.97	Replication, recombination and repair
B	1	0.01	Chromatin structure and dynamics
D	20	0.21	Cell cycle control, cell division, chromosome partitioning
V	20	0.21	Defense mechanisms
T	70	0.74	Signal transduction mechanisms
M	79	0.84	Cell wall/membrane/envelope biogenesis
N	4	0.04	Cell motility
Z	1	0.01	Cytoskeleton
U	26	0.28	Intracellular trafficking, secretion, and vesicular transport
O	75	0.80	Posttranslational modification, protein turnover, chaperones
C	112	1.19	Energy production and conversion
G	114	1.21	Carbohydrate transport and metabolism
E	179	1.90	Amino acid transport and metabolism
F	61	0.65	Nucleotide transport and metabolism
H	110	1.17	Coenzyme transport and metabolism
I	56	0.60	Lipid transport and metabolism
P	111	1.18	Inorganic ion transport and metabolism
Q	48	0.51	Secondary metabolites biosynthesis, transport and catabolism
R	239	2.54	General function prediction only
S	217	2.31	Function unknown
-	7549	80.27	Not in COGs

Table S2. Annotated genes encoding ligninolytic degrading enzymes in strain S6

Feature ID	Function	Size (amino acid)	Organism	% Identity	E-value	Accession No.
	**Peroxidase**					
S6.peg.6773	Catalase (EC:1.11.1.6)	376	*Streptomyces sp.* Dvalaa-19	89	0	WP_029395313.1
S6.peg.7686	Catalase (EC:1.11.1.6)	583	*Streptomyces nanshensis*	100	0	WP_070201881.1
S6.peg.7939	Catalase (EC:1.11.1.6)	166	*Streptomyces cavourensis*	97	7E-116	RBL79238.1
S6.peg.6337	Glutathione peroxidase (EC:1.11.1.9)	162	*Streptomyces fulvissimus* DSM 40593	98	5.00E-113	AGK75755.1
S6.peg.183	Peroxiredoxin (EC:1.11.1.15)	184	*Streptomyces vinaceus*	99	3.00E-133	WP_043246263.1
S6.peg.7259	Peroxiredoxin (EC:1.11.1.15)	161	*Streptomyces sp.* CFMR 7	99	3.00E-112	WP_053560427.1
S6.peg.8810	Thioredoxin-dependent thiol peroxidase (EC:1.11.1.15)	155	*Kitasatospora albolonga*	97	8.00E-106	WP_084746589.1
S6.peg.2209	Catalase/peroxidase HPI (EC:1.11.1.21)	751	*Streptomyces cavourensis*	99	0.00E+00	WP_119823363.1
S6.peg.1953	Dyp-type peroxidase (EC:1.11.1.-)	194	*Streptomyces sp.* ZL-24	100	7.00E-136	WP_103417708.1
S6.peg.8041	Deferrochelatase/peroxidase efeb (EC:1.11.1.-)	390	*Streptomyces nanshensis)*	99	0	WP_070200720.1
	**Dioxygenase**					
S6.peg.1360	Catechol 2,3-dioxygenase (EC:1.13.11.2)	328	*Streptomyces bacillaris*	100	0	WP_112491598.1
S6.peg.7148	Homogentisate 1,2-dioxygenase (EC:1.13.11.5)	353	*Streptomyces nanshensis*	97	0	WP_070200509.1
S6.peg.2405	Tryptophan 2,3-dioxygenase (EC:1.13.11.11)	195	*Streptomyces sp.* Gb14	100	4.00E-114	WP_097970268.1

S6.peg.2711	4-hydroxyphenylpyruvate dioxygenase (EC:1.13.11.27)	381	*Streptomyces cavourensis*	99	0	WP_114931377.1
S6.peg.8438	Extradiol ring-cleavage dioxygenase (EC:1.13.11.-)	46	*Streptomyces nanshensis*	100	2.00E-23	OEV17013.1
S6.peg.4693	4,5-DOPA dioxygenase extradiol (EC:1.13.11.-)	200	*Streptomyces nanshensis*	88	2.00E-109	OEV17013.2
S6.peg.303	Taurine dioxygenase (EC:1.14.11.17)	285	*Streptomyces sp.* CFMR 7	98	0.00E+00	WP_053559843.1
S6.peg.2945	Nitric oxide dioxygenase (EC:1.14.12.17)	398	*Streptomyces sp. Termitarium* T10T6	88	0.00E+00	SCE61787.1
S6.peg.8640	FAD-dependent oxidoreductase	99	*Streptomyces sp.* ZL-24	100	4.00E-46	WP_103416421.1
S6.peg.1033	NAD(P)(also known as FAD-dependent) oxidoreductase	175	*Streptomyces cavourensis*	98	2.00E-113	WP_114930816.1
S6.peg.6514	2-nitropropane dioxygenase	203	*Streptomyces cavourensis*	95	5.00E-105	WP_119825019.1
	**Monooxygenase**					
S6.peg.2995	Amine oxidase	564	*Streptomyces cavourensis*	99	0.00E+00	ATY94699.1
S6.peg.6052	Nitronate monooxygenase (EC:1.13.12.16)	301	*Streptomyces nanshensis*	99	0.00E+00	WP_070204058.1
S6.peg.8542	L-lysine 6-monooxygenase (EC:1.14.13.59)	451	*Streptomyces nanshensis*	99	0.00E+00	WP_070203070.1
S6.peg.8154	Alkanesulfonate monooxygenase (EC:1.14.14.5)	243	*Streptomyces sp.* CBMAI 2042	99	1.00E-174	RLV65111.1
S6.peg.8925	Alkanesulfonate monooxygenase, FMNH(2)-dependent (EC:1.14.14.5)	174	*Streptomyces sp.* CBMAI 2042	94	2.00E-111	RLV65795.1
S6.peg.4879	LLM class flavin-dependent oxidoreductase (EC:1.14.-.-)	134	*Streptomyces sp.* CS090A	100	2.00E-86	WP_109976808.1
S6.peg.7494	4-hydroxyphenylacetate 3-monooxygenase (EC:1.14.14.9)	523	*Amycolatopsis xylanica*	78	0.00E+00	SDW49386.1
S6.peg.119	Dimethylsulfone monooxygenase sfng (EC:1.14.14.35)	393	*Streptomyces sp.* ZL-24	99	0.00E+00	WP_103418753.1

	**Ferroxidase (EC 1.16.3.1)**					
S6.peg.2380	Multicopper oxidase	128	*Streptomyces bacillaris*	98	4.00E-85	WP_112491679.1
S6.peg.2483	Copper oxidase	349	*Streptomyces bacillaris*	99	0.00E+00	WP_112489762.1
S6.peg.4659	Multicopper oxidase	410	*Streptomyces nanshensis*	99	0.00E+00	WP_070203310.1
S6.peg.6734	Bacterioferritin	159	*Streptomyces*	100	5.00E-113	WP_018486665.1
	**Thioredoxin-disulfide reductase (EC 1.8.1.9)**					
S6.peg.5324	NAD(P) (also known as FAD-dependent) oxidoreductase	158	*Streptomyces cavourensis*	100	2.00E-102	WP_114932953.1
S6.peg.7858	Thioredoxin-disulfide reductase	323	*Streptomyces sp.* CFMR 7	99	0.00E+00	WP_053560343.1
	**Superoxide dismutase (EC 1.15.1.1)**					
S6.peg.8145	Nickel superoxide dismutase	131	*Streptomyces*	100	4.00E-92	WP_003966342.1
S6.peg.9033	Superoxide dismutase, Fe-Mn family	267	*Streptomyces*	100	7.00E-106	WP_018487185.1
S6.peg.3942	Hypothethical protein (Superoxide dismutase, Cu-Zn family)	174	*Streptomyces bacillaris*	97	2.00E-117	WP_112492618.1
	**Oxidases**					
S6.peg.2213	Acyl coa oxidase (EC 1.3.3.6)	264	*Streptomyces bacillaris*	100	0.00E+00	WP_112492095.1
S6.peg.2478	Acyl coa oxidase (EC 1.3.3.6)	460	*Streptomyces*	94	0.00E+00	WP_053562175.1
S6.peg.3886	Cytochrome-c oxidase (EC 1.9.3.1) subunit I	429	*Streptomyces purpureus*	92	0.00E+00	WP_019885625.1
S6.peg.6978	Cytochrome-c oxidase (EC 1.9.3.1) subunit I	213	*Streptomyces*	100	5.00E-148	WP_084996031.1
S6.peg.8843	Cytochrome-c oxidase (EC 1.9.3.1) subunit I	223	*Streptomyces bacillaris*	100	1.00E-154	WP_112489509.1
S6.peg.6979	Cytochrome-c oxidase (EC 1.9.3.1) subunit II	322	*Streptomyces*	100	0.00E+00	WP_026237277.1

S6.peg.7964	Cytochrome-c oxidase (EC 1.9.3.1) subunit III	206	*Streptomyces*	100	1.00E-147	WP_029395705.1
S6.peg.7372	Phenylacetyl-coa 1,2-epoxidase subunit PaaA(EC 1.14.13.149)	202	*Streptomyces cavourensis*	100	2.00E-146	WP_119825571.1
S6.peg.7373	Phenylacetyl-coa 1,2-epoxidase subunit PaaA(EC 1.14.13.149)	158	*Streptomyces sp.* Dvalaa-19	100	2.00E-112	WP_093749646.1
S6.peg.7375	Phenylacetyl-coa 1,2-epoxidase subunit PaaC(EC 1.14.13.149)	261	*Streptomyces nanshensis*	98	0.00E+00	WP_070200867.1
S6.peg.2254	Ubiquinol oxidase subunit I (EC 7.1.1.7)	440	*Streptomyces cavourensis*	98	0.00E+00	WP_119827128.1
S6.peg.9047	Ubiquinol oxidase subunit I (EC 7.1.1.7)	503	*Streptomyces* sp.	100	0.00E+00	WP_103418469.1
S6.peg.9046	Ubiquinol oxidase subunit II (EC 7.1.1.7)	333	*Streptomyces* sp.	99	0.00E+00	WP_103418470.1
	**Oxidoreductase**					
S6.peg.8758	NADH-quinone oxidoreductase subunit A (EC 7.1.1.2)	119	*Streptomyces* sp.	100	1.00E-78	WP_018490714.1
S6.peg.2824	NADH-quinone oxidoreductase subunit A (EC 7.1.1.2)	147	*Kitasatospora albolonga*	99	2.00E-94	ARF77313.1
S6.peg.8759	NADH-quinone oxidoreductase subunit B (EC 7.1.1.2)	184	*Streptomycetaceae*	100	6.00E-133	WP_018490715.1
S6.peg.2823	NADH-quinone oxidoreductase subunit B (EC 7.1.1.2)	238	*Streptomyces bacillaris*	100	3.00E-170	WP_112490556.1
S6.peg.8760	NADH-quinone oxidoreductase subunit C (EC 7.1.1.2)	243	*Streptomyces sp.* WAC04770	99	1.00E-176	WP_125621564.1
S6.peg.8761	NADH-quinone oxidoreductase subunit D (EC 7.1.1.2)	440	*Streptomyces* sp.	100	0.00E+00	WP_103416794.1
S6.peg.8762	NADH-quinone oxidoreductase subunit E (EC 7.1.1.2)	212	*Streptomyces sp.* Dvalaa-19	100	1.00E-154	SCE28869.1
S6.peg.9198	NADH-quinone oxidoreductase subunit F (EC 7.1.1.2)	371	*Streptomyces* sp.	100	0.00E+00	WP_070205459.1

S6.peg.9196	NADH-quinone oxidoreductase subunit G (EC 7.1.1.2)	304	*Streptomyces* sp.	100	0.00E+00	WP_103416796.1
S6.peg.3743	NADH-quinone oxidoreductase subunit nuoh (EC 7.1.1.2)	466	*Streptomyces* sp.	100	0.00E+00	WP_093755416.1
S6.peg.1783	NADH-quinone oxidoreductase subunit H (EC 7.1.1.2)	322	*Streptomyces sp.* CFMR 7	100	0.00E+00	WP_053559799.1
S6.peg.3744	NADH-quinone oxidoreductase subunit Nuol (EC 7.1.1.2)	232	*Streptomyces sp.* Dvalaa-19	100	3.00E-171	WP_093755415.1
S6.peg.1782	NADH-quinone oxidoreductase subunit I (EC 7.1.1.2)	194	*Streptomyces sp.* S8	97	2.00E-137	WP_084992946.1
S6.peg.2731	NADH-quinone oxidoreductase subunit J (EC 7.1.1.2)	174	*Streptomyces* sp.	99	4.00E-120	WP_053559827.1
S6.peg.3745	NADH-quinone oxidoreductase subunit J (EC 7.1.1.2)	265	*Streptomyces sp.* Ccalmp-8W	100	4.00E-135	WP_018490723.1
S6.peg.1781	NADH-quinone oxidoreductase subunit J (EC 7.1.1.2)	228	*Streptomyces sp.* WAC04770	99	2.00E-154	WP_125613897.1
S6.peg.1780	NADH-quinone oxidoreductase subunit nuok (EC 7.1.1.2)	140	*Streptomyces nanshensis*	99	6.00E-92	WP_070205428.1
S6.peg.2733	NADH-quinone oxidoreductase subunit L (EC 7.1.1.2)	469	*Streptomyces nanshensis*	100	0.00E+00	WP_070205457.1
S6.peg.2536	NADH-quinone oxidoreductase subunit M (EC 7.1.1.2)	403	*Streptomyces cavourensis*	99	0.00E+00	WP_114932400.1
S6.peg.7621	NADH-quinone oxidoreductase subunit M (EC 7.1.1.2)	399	*Streptomyces sp.* ZL-24	99	0.00E+00	WP_103416831.1
S6.peg.4872	NADH-quinone oxidoreductase subunit M (EC 7.1.1.2)	225	*Streptomyces sp.* ZL-25	89	1.00E-123	WP_103416831.2
S6.peg.2537	NADH-quinone oxidoreductase subunit N (EC 7.1.1.2)	410	*Streptomyces sp.* Dvalaa-19	100	0.00E+00	SCE28773.1
S6.peg.2532	NADH-quinone oxidoreductase subunit N (EC 7.1.1.2)	322	*Streptomyces nanshensis*	99	0.00E+00	WP_070205425.1
S6.peg.4536	NADH-quinone oxidoreductase subunit N (EC 7.1.1.2)	321	*Streptomyces nanshensis*	79	9.00E-167	WP_070205425.1

	**Dehydrogenases**					
S6.peg.83	Zn-dependent alcohol dehydrogenase (EC 1.1.1.1)	393	*Streptomyces* sp.	100	0.00E+00	WP_103416731.1
S6.peg.1690	Zn-dependent alcohol dehydrogenase (EC 1.1.1.1)	281	*Streptomyces nanshensis*	100	0.00E+00	WP_070203349.1
S6.peg.3749	Zn-dependent alcohol dehydrogenase (EC 1.1.1.1)	153	*Streptomyces sp.* SM11	71	1.00E-81	WP_103530902.1
S6.peg.275	Alcohol dehydrogenase, propanol-preferring (EC 1.1.1.1)	392	*Streptomyces nanshensis*	99	0.00E+00	WP_070203095.1
S6.peg.6244	Aryl-alcohol dehydrogenase (EC:1.1.1.90)	114	*Streptomyces nanshensis*	69	1.00E-36	WP_070200123.1
S6.peg.2460	NAD(P)-dependent alcohol dehydrogenase	346	*Streptomyces* sp.	99	0.00E+00	WP_103417068.1
S6.peg.8465	Aldehyde dehydrogenase family protein (EC 1.2.1.3)	346	*Streptomyces sp.* CFMR 7	100	0.00E+00	WP_053559646.1
S6.peg.3475	Aldehyde dehydrogenase family protein (EC 1.2.1.3)	262	*Streptomyces cavourensis*	99	0.00E+00	WP_114931039.1
S6.peg.8336	NAD(P)H: quinone oxidoreductase	175	*Streptomyces* sp.	99	2.00E-177	WP_019762064.1
S6.peg.1565	NAD(P)H dehydrogenase (quinone) (EC 1.6.5.2)	209	*Streptomyces* sp.	100	0.00E+00	WP_053561441.1
S6.peg.1070	NADH dehydrogenase (EC 1.6.99.3)	287	*Streptomyces* sp.	98	0.00E+00	WP_028418913.1
S6.peg.736	NADH dehydrogenase (EC 1.6.99.3)	185	*Streptomyces fulvissimus* DSM 40593	98	1.00E-176	AGK78793.1
S6.peg.2054	NADH dehydrogenase (EC 1.6.99.3)	202	*Streptomyces sp.* Ccalmp-8W	98	0.00E+00	WP_018492545.1
S6.peg.6000	Dehydrogenase	111	*Streptomyces cavourensis*	99	2.00E-91	WP_114930664.1
S6.peg.8076	Acyl CoA dehydrogenase (EC:1.3.-.-)	118	*Streptomyces nanshensis*	99	3.00E-114	WP_070204705.1
S6.peg.1940	Acyl CoA dehydrogenase (EC:1.3.-.-)	541	*Streptomyces* sp.	99	0.00E+00	WP_103419466.1

S6.peg.6600	Acyl CoA dehydrogenase (EC:1.3.-.-)	157	*Streptomyces fulvissimus* DSM 40593	97	6.00E-92	AGK76270.1
S6.peg.6243	NAD(P)-dependent alcohol dehydrogenase (EC:1.1.1.90)	205	*Streptomyces cavourensis*	99	4.00E-86	WP_114933918.1
S6.peg.6367	Aldehyde dehydrogenase	136	*Streptomyces bacillaris*	100	5.00E-88	WP_112491599.1
S6.peg.6367	Aminomuconate-semialdehyde/2-hydroxymuconate-6-semialdehyde dehydrogenase (EC:1.2.1.32 1.2.1.85)	135	*Streptomyces sp.* ZL-24	100	2.00E-87	WP_103416402.1
S6.peg.678	3-hydroxyacyl-coa dehydrogenase (EC:1.1.1.35)	723	*Streptomyces nanshensis*	99	0.00E+00	WP_070201914.1
S6.peg.9091	3-hydroxybutyryl-coa dehydrogenase (EC:1.1.1.157)	282	*Streptomyces* sp.	100	0	WP_019762937.1
S6.peg.334	3-hydroxybutyryl-coa dehydrogenase (EC:1.1.1.157)	286	*Streptomyces* sp.	100	0	WP_026237483.1
S6.peg.2438	3-hydroxybutyryl-coa dehydrogenase (EC:1.1.1.157)	247	*Streptomyces nanshensis*	99	0	WP_070204658.1
S6.peg.5052	3-hydroxybutyryl-coa dehydrogenase (EC:1.1.1.157)	216	*Streptomyces cavourensis*	100	0	WP_114933434.1
S6.peg.5337	3-hydroxybutyryl-coa dehydrogenase (EC:1.1.1.157)	216	*Streptomyces nanshensis*	99	0	WP_070203026.1
	**Reductase**					
S6.peg.8966	Enoyl-(acyl-carrier-protein) reductase (NADH) I (EC 1.3.1.9)	251	*Streptomyces* sp.	100	0.00E+00	WP_018488216.1
S6.peg.4484	NADPH2: quinone reductase (EC:1.6.5.5)	195	*Streptomyces sp.* WAC04770	98	0.00E+00	WP_125618770.1
S6.peg.6532	NADPH2: quinone reductase (EC:1.6.5.5)	195	*Streptomyces fulvissimus*	91	1.00E-104	WP_015610315.1
S6.peg.7858	Thioredoxin reductase (NADPH) (EC:1.8.1.9)	323	*Streptomyces* sp.	100	0.00E+00	WP_029395808.1

S6.peg.7957	Ferredoxin (also known as flavodoxin) ---NADP+ reductase (EC:1.18.1.2, 1.19.1.1)	143	*Streptomyces sp.* Dvalaa-19	98	2.00E-106	SCD43580.1
S6.peg.9124	Ferredoxin (also known as flavodoxin) ---NADP+ reductase (EC:1.18.1.2, 1.19.1.1)	453	*Streptomyces sp.* CBMAI 2042	98	0.00E+00	RLV71272.1
S6.peg.1553	Ferredoxin (also known as flavodoxin) ---NADP+ reductase (EC:1.18.1.2, 1.19.1.1)	379	*Streptomyces sp.* CBMAI 2042	95	0.00E+00	RLV71303.1
	**Trasferase and Hydratase**					
S6.peg.3490	Glutathione S-transferase family protein	446	*Streptomyces bacillaris*	99	0.00E+00	WP_112489928.1
S6.peg.118	Acetyl-coa acyltransferase (EC:2.3.1.16)	608	*Streptomyces sp.* WAC04770	99	0	WP_125616349.1
S6.peg.9078	Enoyl-coa hydratase (EC:4.2.1.17)	187	*Streptomyces sp.* WAC04770	97	2.00E-109	RST19853.1
S6.peg.2567	Enoyl-coa hydratase (EC:4.2.1.17)	269	*Streptomyces cavourensis*	99	0.00E+00	WP_114933394.1
S6.peg.2690	Crotonase(also known as enoyl-coa hydratase) family protein (EC:4.2.1.17)	194	*Streptomyces nanshensis*	96	2.00E-88	WP_070203040.1
S6.peg.4162	Enoyl-coa hydratase (EC:4.2.1.17)	105	*Streptomyces nanshensis*	97	6.00E+65	WP_070203146.1
S6.peg.5546	Enoyl-coa hydratase (EC:4.2.1.17)	231	*Streptomyces nanshensis*	100	4.00E-77	WP_070200156.1
S6.peg.5849	Enoyl-coa hydratase (EC:4.2.1.17)	126	*Streptomyces nanshensis*	100	4.00E-81	WP_070200084.1
S6.peg.5996	Enoyl-coa hydratase (EC:4.2.1.17)	124	*Streptomyces sp.* ZL-24	99	4.00E-75	WP_103421645.1
S6.peg.1941	Acetyl-coa C-acetyltransferase (EC:2.3.1.9)	92	*Streptomyces nanshensis*	98	5.00E-60	WP_070203508.1
S6.peg.1942	Acetyl-coa C-acetyltransferase (EC:2.3.1.9)	293	*Streptomyces nanshensis*	99	0	WP_070203508.2
S6.peg.8855	Acetyl-coa C-acetyltransferase (EC:2.3.1.9)	359	*Streptomyces* sp.	100	0	WP_028417635.1

S6.peg.677	Acetyl-coa C-acetyltransferase (EC:2.3.1.9)	79	*Streptomyces sp.* Cmuel-a718b	100	1.00E-72	SCF60550.1
S6.peg.2248	Acetyl-coa C-acetyltransferase (EC:2.3.1.9)	265	*Streptomyces sp.* Dvalaa-19	100	0	SCD86657.1
S6.peg.2249	Acetyl-coa C-acetyltransferase (EC:2.3.1.9)	88	*Streptomyces sp.* S8	100	1.00E-79	ARI52225.1
S6.peg.3867	Acetyl-coa C-acetyltransferase (EC:2.3.1.9)	388	*Streptomyces sp.* CBMAI 2042	97	0	RLV64571.1
S6.peg.7015	Acetyl-coa C-acetyltransferase (EC:2.3.1.9)	84	*Streptomyces cavourensis*	100	3.00E-75	WP_114933290.1
S6.peg.2831	3-oxoacid coa-transferase subunit A (EC:2.8.3.5)	183	*Streptomyces* sp.	99	3.00E-177	WP_028416463.1
S6.peg.2830	3-oxoacid coa-transferase subunit B (EC:2.8.3.5)	246	*Streptomyces sp.* ZL-24	97	0.00E+00	WP_103417324.1
S6.peg.7828	Histidinol-phosphate aminotransferase (EC:2.6.1.9)	246	*Streptomyces bacillaris*	100	0	WP_112490944.1
S6.peg.8867	Histidinol-phosphate aminotransferase (EC:2.6.1.9)	376	*Streptomyces nanshensis*	100	0	WP_070203175.1
S6.peg.9048	Histidinol-phosphate aminotransferase (EC:2.6.1.9)	73	*Streptomyces* sp.	100	4.00E-66	WP_019767214.1
S6.peg.5562	Aminotransferase	166	*Streptomyces sp.* S8	100	1.00E-163	ARI56717.1
S6.peg.1023	Aconitate hydratase (EC:4.2.1.3)	904	*Streptomyces* sp.	100	0	WP_070202447.1
S6.peg.6887	2-keto-4-pentenoate hydratase/2-oxohepta-3-ene-1,7-dioic acid hydratase	261	*Streptomyces nanshensis*	100	0	WP_070199299.1
	**Cytochrome P450**					
S6.peg.80	Cytochrome P450	108	*Streptomyces sp.* Ccalmp-8W	94	1.00E-96	WP_018491359.1
S6.peg.2869	Cytochrome P450	210	*Streptomyces* sp.	100	0.00E+00	WP_114934335.1
S6.peg.4373	Cytochrome P450	106	*Streptomyces cavourensis*	100	7.00E-98	WP_119827421.1
S6.peg.5470	Cytochrome P450	442	*Streptomyces sp.* CFMR 7	90	0.00E+00	WP_053559918.1

S6.peg.5520	Cytochrome P450	145	*Streptomyces bacillaris*	93	4.00E-118	WP_112489573.1
S6.peg.5966	Cytochrome P450	93	*Streptomyces cavourensis*	99	4.00E-86	WP_119827421.1
S6.peg.7439	Cytochrome P450	455	*Streptomyces* sp.	99	0.00E+00	WP_103417149.1
S6.peg.7758	Cytochrome P450	107	*Streptomyces nanshensis*	100	2.00E-105	WP_070202884.1

Table S3. Putative genes involved in metabolism of central aromatic intermediates

Feature ID	Function	Size (amino acid)	Organism	% Identity	E-value	Accession No.
	**Catechol branch of beta-ketoadipate pathway**					
S6.peg.1360	Catechol 2,3-dioxygenase [EC:1.13.11.2]	328	*Streptomyces bacillaris*	100	0	WP_112491598.1
S6.peg.6883	3-carboxy-cis, cis-muconate cycloisomerase [EC:5.5.1.2]	126	*Streptomyces nanshensis*	99	2.00E-80	WP_070204345.1
S6.peg.2831	3-oxoacid coa-transferase subunit A [EC:2.8.3.5]	183	*Streptomyces* sp.	99	3.00E-177	WP_028416463.1
S6.peg.2830	3-oxoacid coa-transferase subunit B [EC:2.8.3.5]	246	*Streptomyces sp.* ZL-24	97	0.00E+00	WP_103417324.1
S6.peg.2436	3-oxoadipate--succinyl-coa transferase subunit B [EC:2.8.3.5]	278	*Streptomyces sp.* ZL-24	98	0	WP_103417509.1
S6.peg.9253	3-oxoadipate: succinyl-coa transferase	358	*Streptomyces sp.* CFMR 7	100	0	ALC26674.1
	**Homogentisate pathway of aromatic compound degradation**					
S6.peg.7148	Homogentisate 1,2-dioxygenase [EC:1.13.11.5]	353	*Streptomyces nanshensis*	97	0	WP_070200509.1
S6.peg.2711	4-hydroxyphenylpyruvate dioxygenase [EC:1.13.11.27]	381	*Streptomyces cavourensis*	99	0	WP_114931377.1
S6.peg.3671	Fumarylacetoacetase [EC:3.7.1.2]	75	*Streptomyces nanshensis*	100	4.00E-58	WP_070205452.1
S6.peg.4185	Fumarylacetoacetase [EC:3.7.1.2]	342	*Streptomyces sp.* WAC04770	99	0	WP_019764689.1
S6.peg.1023	Aconitate hydratase acna [EC:4.2.1.3]	904	*Streptomyces*	100	0	WP_070202447.1
S6.peg.7828	Histidinol-phosphate aminotransferase [EC:2.6.1.9]	246	*Streptomyces bacillaris*	100	0	WP_112490944.1
S6.peg.8867	Histidinol-phosphate aminotransferase [EC:2.6.1.9]	376	*Streptomyces nanshensis*	100	0	WP_070203175.1

S6.peg.9048	Histidinol-phosphate aminotransferase [EC:2.6.1.9]	73	*Streptomyces*	100	4.00E-66	WP_019767214.1
	**Salicylate and gentisate catabolism**					
S6.peg.6243	NAD(P)-dependent alcohol dehydrogenase [EC:1.1.1.90]	205	*Streptomyces cavourensis*	99	4.00E-86	WP_114933918.1
S6.peg.6244	Aryl-alcohol dehydrogenase [EC:1.1.1.90]	114	*Streptomyces nanshensis*	69	1.00E-36	WP_070200123.1
S6.peg.3671	Fumarylacetoacetase [EC:3.7.1.2]	75	*Streptomyces nanshensis*	100	4.00E-58	WP_070205452.1
S6.peg.4185	Fumarylacetoacetase [EC:3.7.1.2]	342	*Streptomyces sp.* WAC04770	99	0	WP_019764689.1
S6.peg.6887	2-keto-4-pentenoate hydratase/2-oxohepta-3-ene-1,7-dioic acid hydratase	261	*Streptomyces nanshensis*	100	0	WP_070199299.1
S6.peg.3366	2-hydroxyhepta-2,4-diene-1,7-dioate isomerase	218	*Streptomyces nanshensis*	100	2.00E-157	OEV19985.1

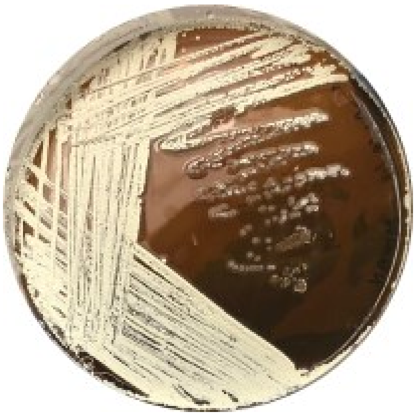

Figure S1. Growth of isolated strain S6 in solid media containing alkali lignin as sole carbon source

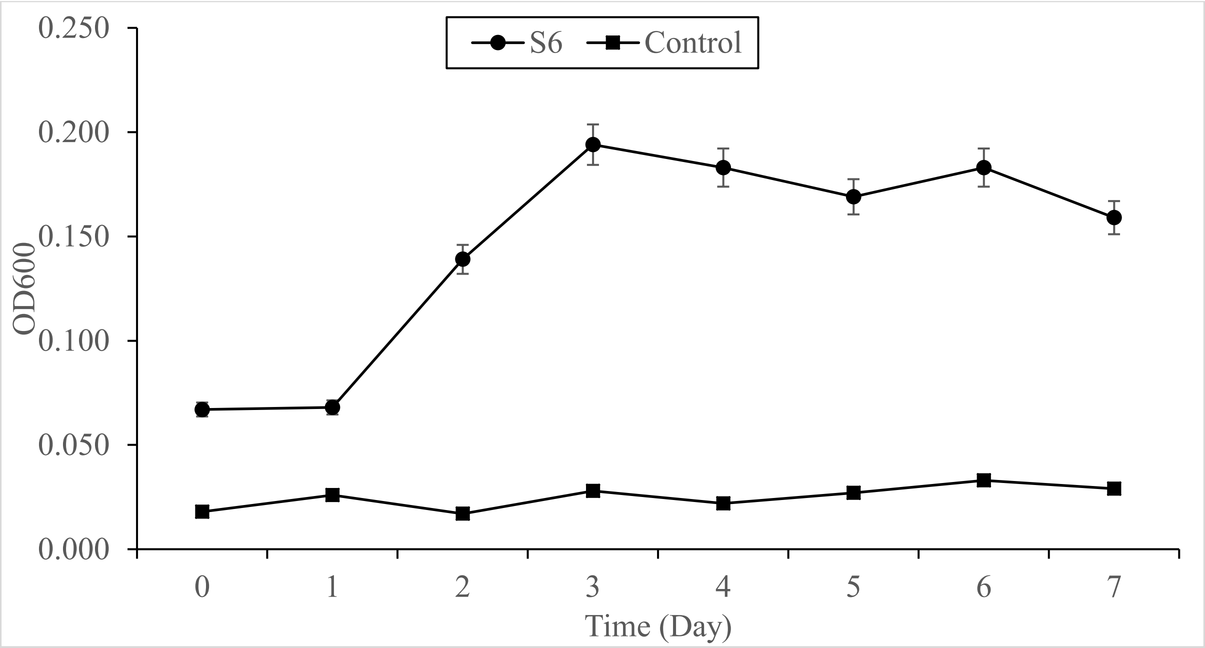

Figure S2. Growth curve of strain S6 in liquid medium containing alkali lignin as sole carbon source. A non-inoculated control is shown with a square marker line.

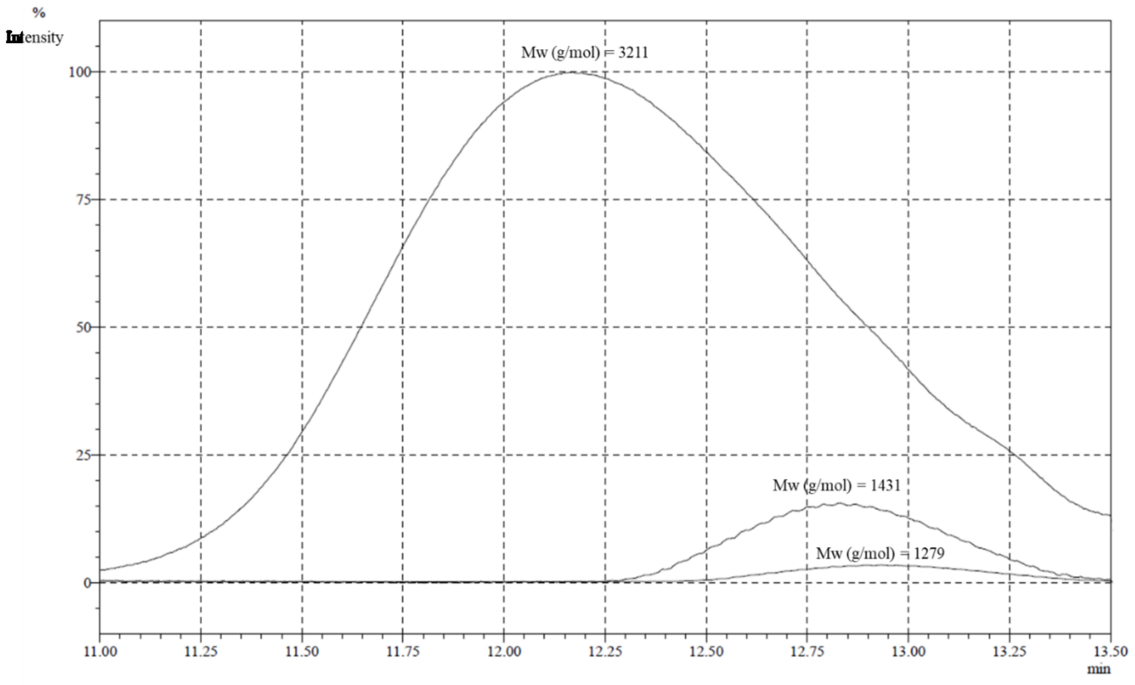

Figure S3. GPC chromatogram data of the isolated strain S6 at (i) Control (untreated KL without S6), (ii) day 3 and (iii) day 7.

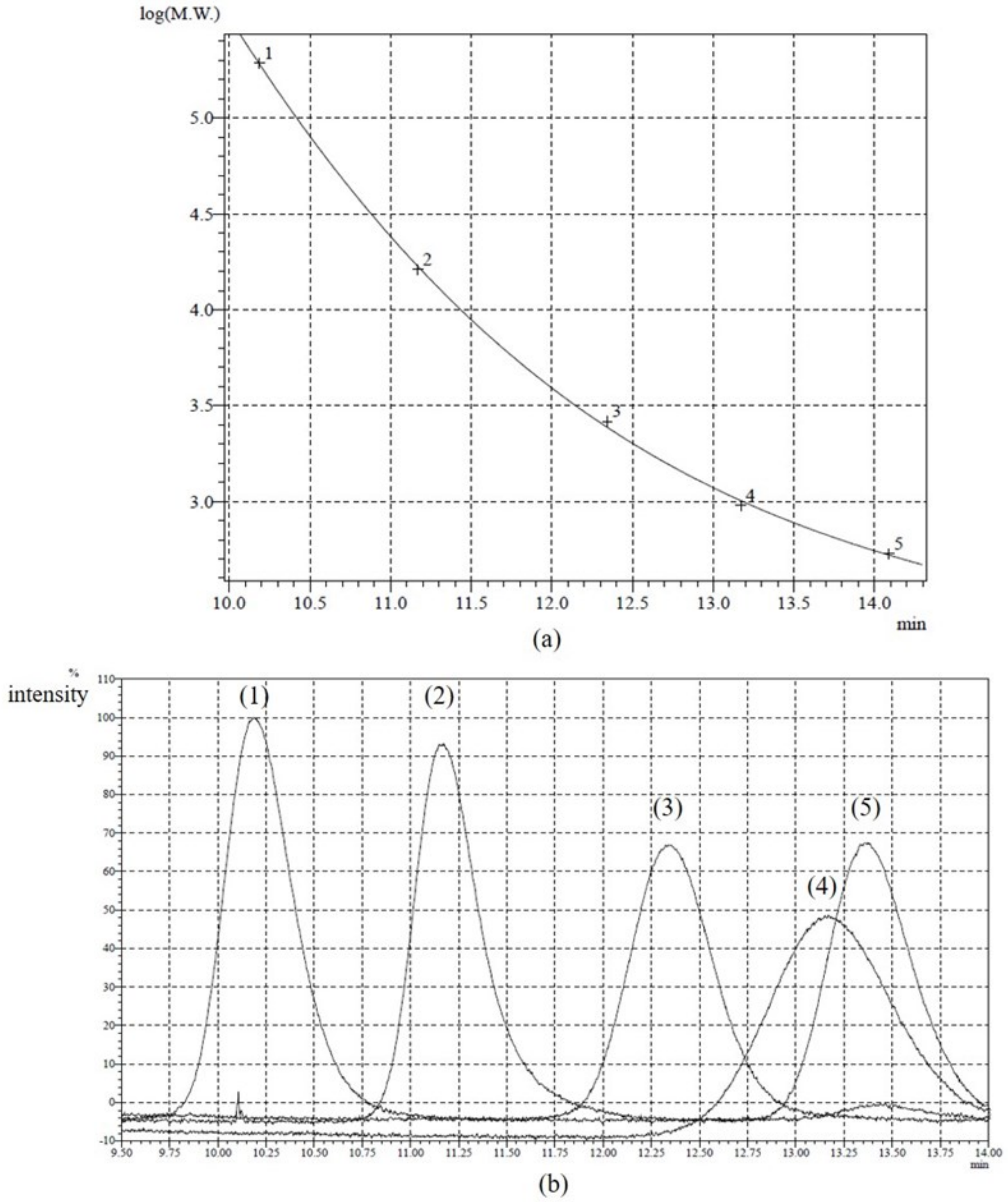

(b)

Figure S4: GPC chromatogram illustrating the lignin standard markers of the (a) calibration line and (b) molecular weight (1): 193 000 Da, (2): 16 200 Da, (3): 2 620 Da, (4): 953 Da, (5): 533 Da

A.

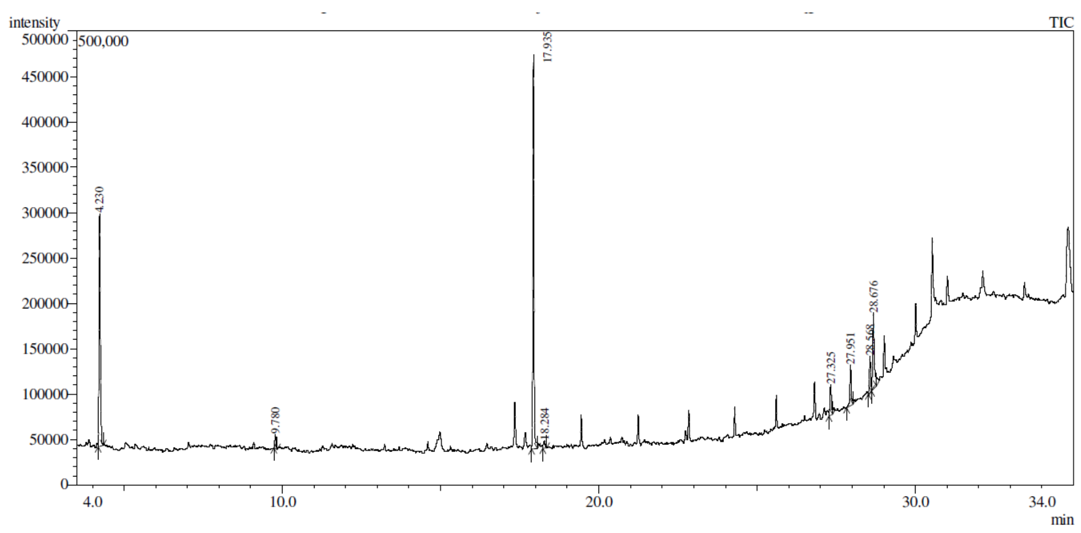

intensity
TIC
500,000
500000
450000
400000
350000
300000
250000
200000
150000
100000
50000
0
4.230
9.780
17.935
18.284
27.325
27.951
28.568
28.676
4.0
10.0
20.0
30.0
34.0
min

B.

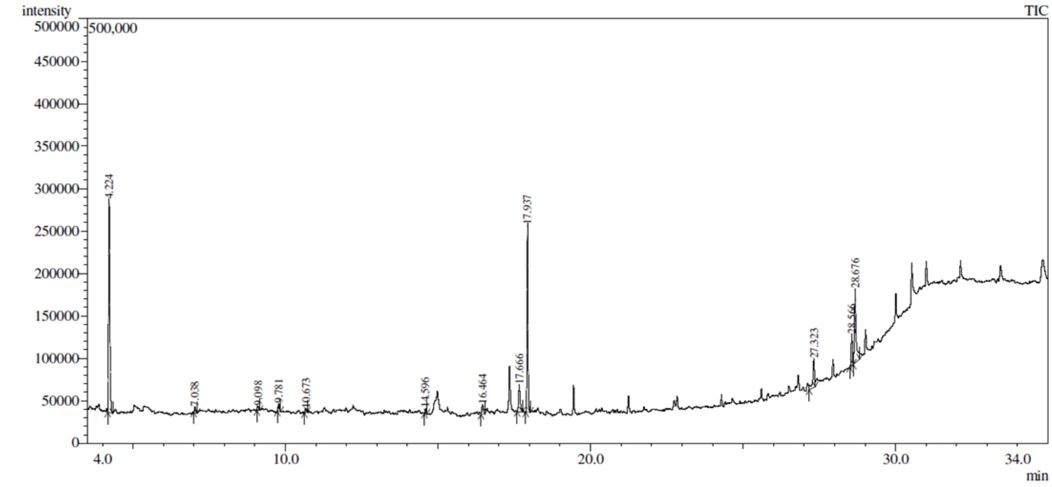

(a)

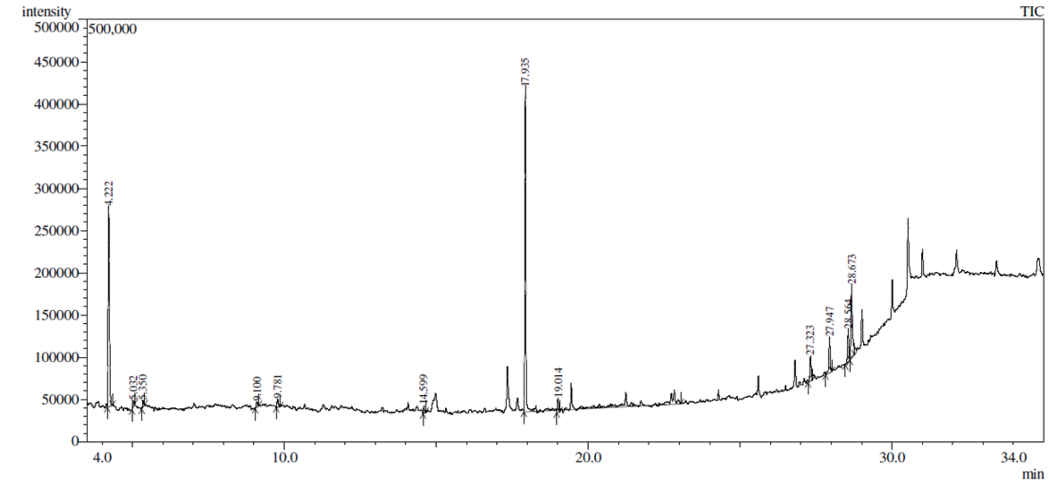

(b)

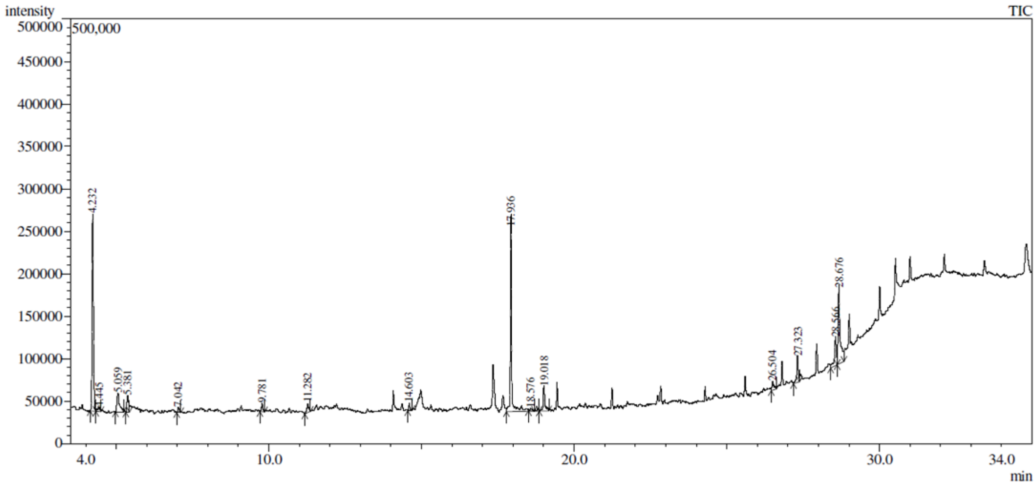

(c)

Figure S5. A: TIC of metabolites produced by control (untreated samples) B: TIC of metabolites produced by S6 from KL degradation (a) day 0, (b) day 3, and (c) day 7
